# Safety and Efficacy of the Prostar XL Vascular Closing Device for Percutaneous Closure of Large Arterial Access Sites

**DOI:** 10.1155/2013/875484

**Published:** 2013-01-14

**Authors:** Christoph Thomas, Volker Steger, Stefan Heller, Martin Heuschmid, Dominik Ketelsen, Claus D. Claussen, Klaus Brechtel

**Affiliations:** ^1^Diagnostic and Interventional Radiology, Department of Radiology, University of Tübingen, Hoppe-Seyler-Straße 3, 72076 Tübingen, Germany; ^2^Department of Thoracic, Cardiac and Vascular Surgery, University of Tübingen, Hoppe-Seyler-Straße 3, 72076 Tübingen, Germany

## Abstract

*Purpose*. The purpose of this study is to retrospectively evaluate the efficacy and safety of the Prostar XL device for percutaneous large access site closure in an unselected patient and operator collective. *Materials and Methods*. All patients (*n* = 50) who had received percutaneous vascular closing with the Prostar XL device in our institution with follow-up data of at least 6 months were retrospectively included. Primary (freedom from surgical conversion) and continued (freedom from groin surgery in further course) technical success and major (deviations from expected outcome requiring surgery) and minor (other deviations from expected outcome) complications were assessed. Success and complications rates were correlated with delivery system size (Mann-Whitney Rank Sum Tests) and operator experience (paired samples *t*-test). *Results*. Rates of primary and continued technical success as well as major and minor complications were 93.6%, 89.7%, 10.3%, and 10.3% (groin based) and 90.0%, 84.0%, 16.0%, and 16.0% (patient based), respectively. No correlation of success and complications rate was found with delivery system sizes and operator experience. *Conclusions*. Application of the Prostar XL device for percutaneous closure of large arterial access sites is safe with a relatively high rate of technical success and low rate of major complications. Sizes of the delivery systems and the experience of the operator did not influence the results.

## 1. Introduction

Within the recent decade, the demand for transcatheter interventions has been increasing in both low-profile (up to 10 F) and high-profile systems (10–25 F). While a large number of vascular closing products, such as collagen plugs, clips, and sealing agents, exist for the closure of smaller vessel access sites of up to 8 F, these devices are not sufficient for large artery access [1]. 

With ongoing technical development, improved flexibility, and lower profiles, the interest in percutaneously implantable devices has been increased in particular in elder patients with limited cardiovascular function and other comorbidities and high perioperative mortality. This is especially true for infrarenal and thoracic endovascular aneurysm repair (EVAR and TEVAR) as well as for transcatheter aortic valve implantation (TAVI). The number of these patients can be expected to grow in the near future due to the lower perioperative mortality of percutaneous procedures [2]. EVAR, TEVAR, and TAVI require large arterial access with sheaths of typically 14–25 French (F) [3]. Due to the lack of appropriate closure devices, surgical cutdown of the groin was necessary in the past in order to achieve sufficient hemostasis when using larger devices [4]. However, cutdown may lengthen the intervention time and is associated with possible complications like hematoma, seroma, lymphoceles, and infection and leads to a significantly longer duration of the hospital stay [5].

Decreasing profiles of the delivery systems and the development of suture-mediated arterial closure devices have initiated a transition to completely percutaneous repair of aortic aneurysms [5]. The Prostar XL device (Abbott Vascular, Santa Clara, CA, USA) is currently the only FDA- and CE-approved percutaneous vascular closing device for closure of large-size femoral artery puncture sites (8.5–10 F) [6]. Two other suture-mediated closing systems exist for smaller access sites (5–8 F, PerClose and Proglide, Abbott Vascular). For larger access sites, as necessary for EVAR, TEVAR, and TAVI, these devices have to be used off-label in terms of a so-called “preclosing” procedure [7, 8].

Several studies evaluating the efficacy of this device already exist in the literature [4–21]. However, in the majority of these studies, only experienced users were allowed to contribute to the database, which in combination with the prospective nature of most studies might have posed a bias. 

The aim of this study was to retrospectively evaluate the efficacy and safety of percutaneous vascular access site closure using the Prostar XL device in an unselected patient and operator collective at a single academic institution without excluding cases due to limited operator experience.

## 2. Materials and Methods

The permission to perform this retrospective study was obtained from the institutional review board with waiver of informed consent. 

A query of the radiological information system (RIS) was performed to retrospectively identify all patients who had received percutaneous vascular closure using the Prostar XL device (Abbott Vascular) in our institution until 04/2011. Two patients with ruptured infrarenal aortic aneurysms passed away intraoperatively due to reasons not associated with the access sites, and one patient who was lost to followup was excluded. All other patients who were returned by the query were included into the study. 

Radiological and clinical patient documentation regarding the intervention and the following hospital stay were reviewed for each patient. Furthermore, follow-up imaging and clinical documentation for a period of at least six months after the intervention were reviewed. The following data were recorded: demographic data, the type of intervention, the sizes of the used delivery systems and/or access sheaths, the incidence of groin bleeding after performing the vascular closure, leading to prolonged manual postinterventional compression (defined as compression time of 10 to 60 minutes) or surgical conversion, and the incidence of access site pseudoaneurysms, hematoma, or other access-related groin complications up to six months after the intervention. 

**Figure 1:**
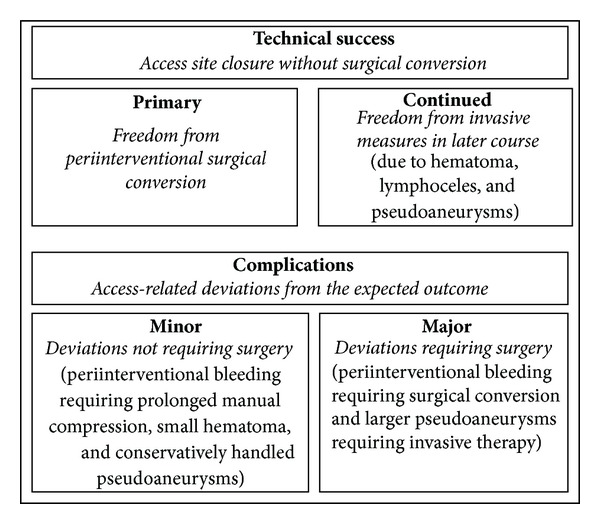
Overview over outcome measures (technical success and complications).

Outcome measures were rates of primary and continued technical success (Figure 1). Primary technical success was defined as successful percutaneous closure of the access site without need for surgical conversion [7]. In concordance to most other published studies, the need for prolonged manual compression was not regarded as primary technical failure [7, 8, 14]. Continued technical success was defined as freedom from further surgical and endovascular measures due to hematoma, lymphoceles, or pseudoaneurysms during followup [7]. 

**Figure 2:**
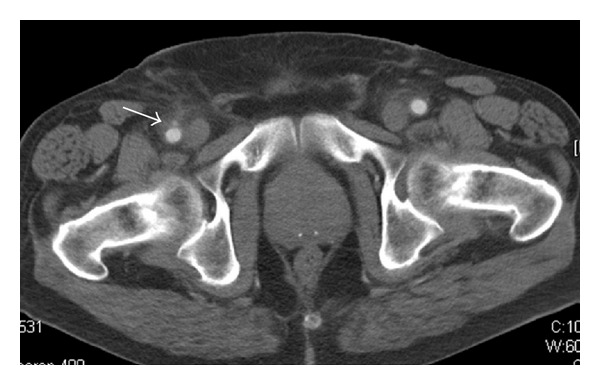
Small aneurysma spurium of the right common femoral artery (arrow) which was detected in CT imaging three days after percutaneous EVAR. The aneurysm could be treated successfully with compression.

**Figure 3:**
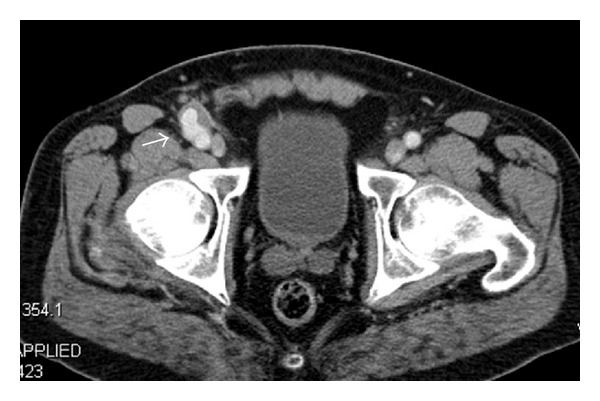
Larger aneurysma spurium of the right common femoral artery (arrow) with a wide neck which was detected by CT imaging three months after percutaneous EVAR, requiring surgical cutdown.

Furthermore, all complications that were related to the access procedure were recorded. Minor complications were defined as access-related deviations from the expected outcome that did not require surgical intervention, including prolonged manual compression, small hematoma, and pseudoaneurysms which could be handled with conservative measures (Figure 2) [8]. Major complications required immediate or prolonged surgical or medical intervention such as periinterventional bleeding with following surgical conversion or larger pseudoaneurysms, requiring groin surgery (Figure 3) [8]. 

Results are given as groin-based and patient-based. Furthermore, technical success rates and incidence of complications are given per delivery system size. 

Mann-Whitney Rank Sum Tests were applied to test for significant differences between the incidence of complications with smaller (≤16 F) and larger (≥18 F) sheath sizes. The effect of the operator experience is assessed visually by plotting successful use and complicating events separately for each of the three operators who used the device in our department during the analyzed time interval. For operator 1 who performed most of the cases (*n* = 57 groins), a statistical evaluation to assess whether a learning curve exists is performed by dividing the cases into two groups, consisting in the first 28 and in the last 29 groins, respectively. The frequency of prolonged compression, surgical conversion, and pseudoaneurysms is assessed in both groups and compared using a paired samples *t*-test.

## 3. Results

### 3.1. Demographics

Patient demographics are summarized in Table 1. Between 12/2008 and 04/2011, the Prostar XL device was applied in 50 patients (49 males, mean age 79 years) and 78 vessels, respectively. Procedures were abdominal EVAR (*n* = 40), TEVAR (*n* = 5), iliac aneurysm repair (*n* = 4), and stent assisted coiling of an aortic aneurysm (*n* = 1). Mean size of the used access sheaths and delivery systems was 16.9 F (range 8–25 F). Periprocedural complications are summarized in Table 2. 

### 3.2. Technical Success and Complications

#### 3.2.1. Groin-Based Analyses

In 6 groins (7.7%), prolonged manual compression after vascular closing due to bleeding was necessary. In 5 groins (6.4%), surgical conversion due to gross bleeding during the application of the sutures (*n* = 1) and after the removal of the delivery system (*n* = 4) was necessary. In all of these cases, hemostasis could be reached by surgical repair. Primary technical success was therefore achieved in 93.6% of all groins. 

In 5 cases (6.4%), pseudoaneurysms were detected up to three months after the intervention. After this time period, none of the patients experienced further complications. Two aneurysms could be managed conservatively, and two aneurysms required surgical treatment, leading to a continued technical success of 89.7%. The rate of major and minor complications was 10.3% each.

#### 3.2.2. Patient-Based Analyses

In a patient-based evaluation, 6 patients (12.0%) required prolonged manual compression due to postinterventional bleeding, and 5 patients (10.0%) required immediate surgical cutdown. Pseudoaneurysms occurred in 5 patients (10%); in two patients (4.0%) surgical treatment was necessary, leading to a patient-based primary technical success of 90% and a continued technical success of 84%. The patient-based rate of major and minor complications was 16.0% each.

#### 3.2.3. Delivery System Size

Technical success rates and complications per delivery system size are given in Table 3. Mann-Whitney Rank Sum Tests did not reveal statistically relevant differences in the incidence of complications between smaller and larger sheath sizes (Table 3).

#### 3.2.4. Operator-Based Analyses

**Figure 4:**
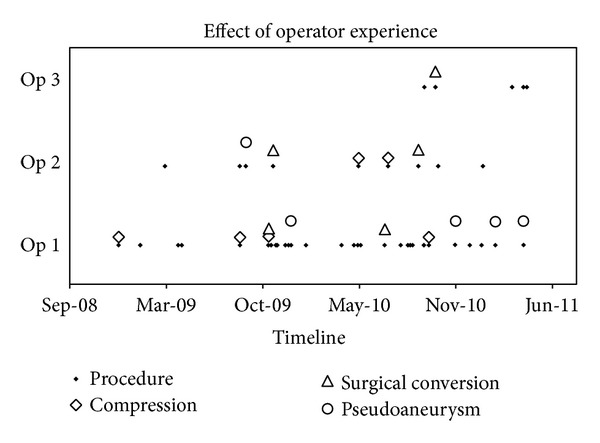
Number of procedures and complications for each operator displayed over time. Op 1–3: single operators; compression: need for postinterventional manual compression of 5–60 minutes; surgical conversion: incidence of groin bleeding, requiring surgical conversion; pseudoaneurysm: incidence of pseudoaneurysm.

Figure 4 and Table 4 show the successful applications and the complications of each operator along a timeline. Complicating events are distributed relatively even over time without indication of a learning curve. The paired samples *t*-test did not reveal significant differences regarding complications and the incidence of pseudoaneurysms in the first 28 and the last 29 patients (*P* = 0.8614). However, it has to be remarked that the operators did not work fully independently from each other during their first cases but benefitted from each other's experiences and from the advice of company representatives.

## 4. Discussion

The interest of complete percutaneous interventions with high profile systems has increased recently [2]. In EVAR, the technical development of the so-called “low-profile” systems not larger than 20 F contributed to the interest in the transition from surgical cutdown to suture-mediated devices. Even in larger artery access, as used in TAVI and TEVAR, the off-label preclosing technique can be safely applied by using a single or even multiple devices [3]. Recent published results (works from 2006–2011) report on groin-based primary technical success rates of 83%–100% [3, 8–12, 14–16]. With a rate of groin-based primary technical success of 94% and a rate of continued technical success of 90%, the results in our study are well within the range of these previously published results. Our results confirm that the use of the Prostar XL device is safe and poses a valid alternative to primary surgical cutdown. 

Previous comparative studies have confirmed that completely percutaneous EVAR leads to a significant reduction of intervention time and time to ambulation [3, 5, 7, 14, 15]. However, since postinterventional hemorrhage requiring surgical cutdown occurred in 10% of the patients and thus still poses a relevant procedural risk, the procedure should only be performed in settings where a rapid surgical support is available in case of device failure. 

In contrast to another study by Starnes et al. [8], we did not find an increased rate of complications for larger delivery systems sizes; this finding is in concordance with the results of Etezadi et al. [5] who did not find a significant correlation between complications and increased sheath sizes; however, this group only evaluated sheath sizes of up to 18 F. 

Heavy vessel calcification and obesity are the main risk factors for device failure and major complications leading to the requirement of surgical conversion as shown by Eisenack et al. [21]. This is due to the technical principle of the suture device which is based on retrograde puncture of the arterial wall with nitinol needles. The success of this step will be clearly limited when heavily calcified plaques have to be crossed. By avoiding the chance of complications in patients with heavily calcified anterior portions of the common femoral arteries, these patients are routinely excluded from completely percutaneous EVAR in our department. Thus, no heavy vessel calcification within the anterior vessel wall occurred within the analyzed cohort. However, heavily calcified plaques could be observed within the dorsal portion of the common femoral artery but did not contribute to device failure (data not shown). 

The negative effect of morbid obesity has been documented by the groups of Teh et al. [12] and Starnes et al. [8] but could not be confirmed in the study of Etezadi et al. [5]. In this study, the influence of obesity onto the success rate was not evaluated due to limited clinical documentation in this respect. 

To our best knowledge, this is the first retrospective study assessing the effect of operator experience onto the success rate. Visual and statistical assessments allow the observation that there is no accumulation of complications during the first procedures, which might be partly explained with assistance which is offered to inexperienced users by company representatives and colleagues. 

This study had certain limitations. First of all, the number of patients which were included is limited since this is a single center study. The design of the study was retrospective, which decreases the power of the results. On the other hand, since the interventionalists were not aware that their results would be included into a study, operator bias can be regarded as very low.

In conclusion, the use of the Prostar XL device for percutaneous closure of large arterial access sites is safe with a relatively high rate of technical success and low rate of major complications. In this limited cohort, sizes of the delivery system and the experience of the operator did not influence the results.

## Figures and Tables

**Table 1:** Overview over patients and procedures.

	*n* (range)	%
Patients	50	
Male sex	49	98%
Age	70 (28–69)	
EVAR	40	80%
TEVAR	5	10%
Iliac prosthesis	4	8%
Stent-assisted coiling of aneurysm	1	2%

**Table 2:** Overview over technical success and complications. PA: pseudoaneurysm. Technical success and complications are defined in the text and in Figure 1.

	Groin-based (*n* = 78)		Patient-based (*n* = 50)	
****	*n*	%	*n*	%
Manual compression	6	7.7%	6	12.0%
Surgical conversion	5	6.4%	5	10.0%
PA (conservative)	2	2.6%	2	4.0%
PA (surgical)	3	3.8%	3	6.0%
Primary technical success	73	93.6%	45	90.0%
Continued technical success	70	89.7%	42	84.0%
Major complications	8	10.3%	8	16.0%
Minor complications	8	10.3%	8	16.0%

**Table 3:** Technical success (given in %) and number of complications at all 78 access sites based on sheath size. Additionally, *P* values of Mann-Whitney Rank Sum Tests comparing smaller (≤16) and larger (≥18) access sites are given.

Sheath size (Fr)	*n*	Compr.	Surgical conversion	PA (cons.)	PA (surg.)	Primary technical success	Continued technical success	Major compl.	Minor compl.
8	1					100%	100%	0%	0%
10	2	1				100%	100%	0%	50%
12	1					100%	100%	0%	0%
14	28	3	3	1		89%	89%	11%	14%
16	8				2	100%	75%	25%	0%
18	9		1			89%	89%	11%	0%
20	24	2	1	1	1	96%	92%	8%	17%
22	1					100%	100%	0%	0%
24	3					100%	100%	0%	0%
25	1					100%	100%	0%	0%

≤16	40	4	3	1	2	93%	88%	13%	13%
≥18	38	2	2	1	1	95%	92%	8%	11%
*P*		0.442	0.697	0.538	0.599	n.a.	n.a.	n.a.	n.a.

Compr.: need for prolonged compression; PA (cons.): pseudoaneurysm with conservative management; PA (surg.): pseudoaneurysm with surgical management; major/minor compl.: major/minor complications.

**Table 4:** Pooled numbers of complications in the first 28 and the last 29 groins operated on by operator 1. Paired samples *t*-test did not reveal a significant difference between both groups.

	Groins 1–28	Groins 29–57
Prolonged compression (*n*)	3	1
Surgical conversion (*n*)	1	1
PA, conservative management (*n*)	0	1
PA, surgical management (*n*)	1	2
